# 2,4-Dinitro-1-phenoxy­benzene

**DOI:** 10.1107/S1600536810001911

**Published:** 2010-01-20

**Authors:** Zhen-Ting Du, Yan Xu, Hong-Rui Yu, Yong Li

**Affiliations:** a College of Science, Northwest A&F University, Yangling Shaanxi 712100, People’s Republic of China

## Abstract

The title compound, C_12_H_8_N_2_O_5_, was obtained by the reaction of 1-chloro-2,4-dinitro­benzene and phenol in the presence of potassium carbonate. The nitro-substituted benzene ring lies on a mirror plane, with one NO_2_ group in the same plane and the other disordered across this plane. The phenoxy­benzene unit is placed perpendicular to this mirror, resulting in an exact orthogonal relationship between the phenyl and benzene rings in the mol­ecule. The crystal packing exhibits no significantly short inter­molecular contacts.

## Related literature

For the synthesis of the title ether, see: Williamson (1852 ▶); Paul & Gupta (2004 ▶). For a related structure, see: Gopal *et al.* (1980 ▶).
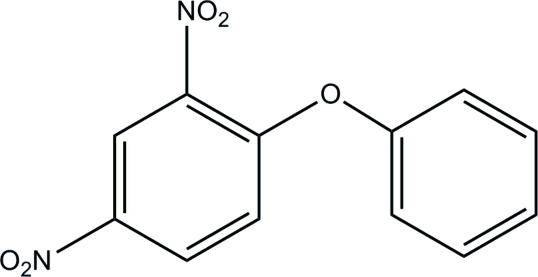


## Experimental

### 

#### Crystal data

C_12_H_8_N_2_O_5_
*M* *_r_* = 260.20
Orthorhombic, 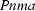

*a* = 21.012 (13) Å
*b* = 6.917 (4) Å
*c* = 8.211 (5) Å
*V* = 1193.4 (12) Å^3^
*Z* = 4
Mo *K*α radiation
μ = 0.12 mm^−1^
*T* = 298 K
0.50 × 0.47 × 0.45 mm

#### Data collection

Bruker SMART APEX CCD area-detector diffractometer
Absorption correction: multi-scan (*SADABS*; Sheldrick, 1996 ▶) *T*_min_ = 0.945, *T*_max_ = 0.950
5246 measured reflections
1150 independent reflections
639 reflections with *I* > 2σ(*I*)
*R*_int_ = 0.069

#### Refinement

*R*[*F* ^2^ > 2σ(*F* ^2^)] = 0.066
*wR*(*F* ^2^) = 0.220
*S* = 1.03
1150 reflections
117 parameters
16 restraints
H-atom parameters constrained
Δρ_max_ = 0.30 e Å^−3^
Δρ_min_ = −0.20 e Å^−3^

### 

Data collection: *SMART* (Siemens, 1996 ▶); cell refinement: *SAINT* (Siemens, 1996 ▶); data reduction: *SAINT*; program(s) used to solve structure: *SHELXS97* (Sheldrick, 2008 ▶); program(s) used to refine structure: *SHELXL97* (Sheldrick, 2008 ▶); molecular graphics: *SHELXTL* (Sheldrick, 2008 ▶); software used to prepare material for publication: *SHELXTL*.

## Supplementary Material

Crystal structure: contains datablocks I, global. DOI: 10.1107/S1600536810001911/bh2266sup1.cif

Structure factors: contains datablocks I. DOI: 10.1107/S1600536810001911/bh2266Isup2.hkl

Additional supplementary materials:  crystallographic information; 3D view; checkCIF report

## Figures and Tables

**Fig. 1.**
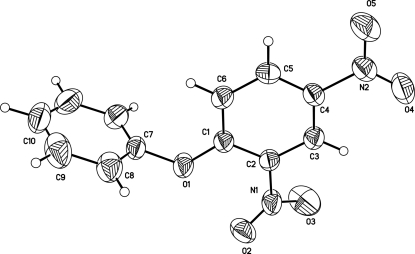
ORTEP drawing of the title complex with atomic numbering scheme and thermal ellipsoids at 30% probability level. Disordered atoms O2 and O3 generated by symmetry x, 1/2-y, z (m plane) have been omitted. Unlabelled atoms in the phenyl ring are generated by symmetry x, 1/2-y, z.

## supplementary crystallographic information

### Comment

One of the most common procedures for the synthesis of ethers was originally introduced by Williamson, and involves the reaction of alkoxides with alkyl halides (Williamson, 1852). This method has been known for nearly 170 years, and remains a very useful transformation in organic synthesis (Paul & Gupta, 2004).

In this paper, we present a new crystal structure, 2,4-dinitro-1-phenoxybenzene, (I), which was synthesized by the reaction of 1-chloro-2,4-dinitrobenzene and phenol, in the presence of potassium carbonate (see *Experimental*).

In (I) (Fig. 1), the bond lengths and angles are normal and comparable to those observed in related compounds (*e.g*. Gopal *et al.*, 1980). The angle between the benzene and the phenyl rings is 90° by symmetry. In the crystal structure, no significantly short intermolecular contacts are observed.

### Experimental

1-Chloro-2,4-dinitrobenzene (10 mmol), potassium carbonate (20 mmol), phenol (6 mmol), and 20 ml of acetone were mixed in a 50 ml flask. After stirring for 2 h. at 373 K, the crude product was obtained. Crystals were obtained by recrystallization from *n*-hexane/ethyl acetate. Elemental analysis: calculated for C_12_H_8_N_2_O_5_: C 55.39, H 3.10, N 10.77%; found: C 55.21, H 3.18, N 10.59%.

### Refinement

All H atoms were positioned geometrically, with C—H = 0.93 Å, and refined as riding, with *U*_iso_(H) = 1.2*U*_eq_(carrier C). The refinement was carried-out using a model which includes 16 restraints: in order to converge to a sensible geometry for the phenyl ring mirrored in the symmetry plane, bond lengths C7—C8, C8—C9 and C9—C10 were restrained to 1.39 (1) Å. For the disordered nitro group, bond lengths N1—O2 and N1—O3 were averaged, and atoms N1, O2 and O3 were restrained to have similar displacement parameters.

### Crystal data

**Table:**

C_12_H_8_N_2_O_5_	*F*(000) = 536
*M**_r_* = 260.20	*D*_x_ = 1.448 Mg m^−^^3^
Orthorhombic, *P**n**m**a*	Mo *K*α radiation, λ = 0.71073 Å
Hall symbol: -P 2ac 2n	Cell parameters from 1105 reflections
*a* = 21.012 (13) Å	θ = 2.7–21.4°
*b* = 6.917 (4) Å	µ = 0.12 mm^−^^1^
*c* = 8.211 (5) Å	*T* = 298 K
*V* = 1193.4 (12) Å^3^	Block, red
*Z* = 4	0.50 × 0.47 × 0.45 mm

### Data collection

**Table:**

Bruker SMART APEX CCD area-detector diffractometer	1150 independent reflections
Radiation source: fine-focus sealed tube	639 reflections with *I* > 2σ(*I*)
graphite	*R*_int_ = 0.069
φ and ω scans	θ_max_ = 25.0°, θ_min_ = 1.9°
Absorption correction: multi-scan (*SADABS*; Sheldrick, 1996)	*h* = −23→25
*T*_min_ = 0.945, *T*_max_ = 0.950	*k* = −8→8
5246 measured reflections	*l* = −5→9

### Refinement

**Table:**

Refinement on *F*^2^	Secondary atom site location: difference Fourier map
Least-squares matrix: full	Hydrogen site location: inferred from neighbouring sites
*R*[*F*^2^ > 2σ(*F*^2^)] = 0.066	H-atom parameters constrained
*wR*(*F*^2^) = 0.220	*w* = 1/[σ^2^(*F*_o_^2^) + (0.1048*P*)^2^ + 0.4983*P*] where *P* = (*F*_o_^2^ + 2*F*_c_^2^)/3
*S* = 1.03	(Δ/σ)_max_ < 0.001
1150 reflections	Δρ_max_ = 0.30 e Å^−^^3^
117 parameters	Δρ_min_ = −0.20 e Å^−^^3^
16 restraints	Extinction correction: *SHELXL97* (Sheldrick, 2008), Fc^*^=kFc[1+0.001xFc^2^λ^3^/sin(2θ)]^-1/4^
0 constraints	Extinction coefficient: 0.023 (6)
Primary atom site location: structure-invariant direct methods	

### Fractional atomic coordinates and isotropic or equivalent isotropic displacement parameters (Å^2^)

**Table:**

	*x*	*y*	*z*	*U*_iso_*/*U*_eq_	Occ. (<1)
N1	0.4600 (2)	0.2500	0.9620 (5)	0.0970 (18)	
N2	0.5869 (2)	0.2500	0.4695 (6)	0.0782 (13)	
O1	0.35033 (13)	0.2500	0.7731 (4)	0.0844 (13)	
O2	0.4181 (2)	0.3065 (14)	1.0352 (6)	0.120 (3)	0.50
O3	0.5041 (3)	0.1576 (11)	1.0291 (7)	0.146 (3)	0.50
O4	0.63351 (19)	0.2500	0.5549 (6)	0.1135 (17)	
O5	0.5895 (2)	0.2500	0.3233 (6)	0.1127 (16)	
C1	0.4064 (2)	0.2500	0.6926 (6)	0.0600 (13)	
C2	0.4620 (2)	0.2500	0.7843 (5)	0.0597 (13)	
C3	0.5208 (2)	0.2500	0.7124 (6)	0.0641 (13)	
H3	0.5577	0.2500	0.7752	0.077*	
C4	0.5240 (2)	0.2500	0.5464 (6)	0.0590 (12)	
C5	0.4704 (2)	0.2500	0.4509 (6)	0.0633 (13)	
H5	0.4738	0.2500	0.3380	0.076*	
C6	0.4115 (2)	0.2500	0.5237 (6)	0.0662 (14)	
H6	0.3750	0.2500	0.4598	0.079*	
C7	0.2935 (2)	0.2500	0.6851 (6)	0.0691 (15)	
C8	0.26597 (18)	0.4227 (7)	0.6495 (5)	0.0960 (14)	
H8	0.2854	0.5386	0.6781	0.115*	
C9	0.2084 (2)	0.4202 (10)	0.5698 (6)	0.130 (2)	
H9	0.1891	0.5362	0.5410	0.155*	
C10	0.1795 (3)	0.2500	0.5328 (9)	0.139 (4)	
H10	0.1400	0.2500	0.4820	0.167*	

### Atomic displacement parameters (Å^2^)

**Table:**

	*U*^11^	*U*^22^	*U*^33^	*U*^12^	*U*^13^	*U*^23^
N1	0.055 (3)	0.182 (5)	0.054 (3)	0.000	−0.006 (2)	0.000
N2	0.071 (3)	0.089 (3)	0.074 (3)	0.000	0.015 (3)	0.000
O1	0.053 (2)	0.149 (4)	0.0508 (19)	0.000	−0.0026 (16)	0.000
O2	0.089 (3)	0.209 (10)	0.062 (3)	0.054 (4)	−0.001 (2)	−0.017 (4)
O3	0.146 (4)	0.223 (8)	0.070 (3)	0.075 (5)	−0.013 (3)	0.022 (4)
O4	0.061 (2)	0.181 (5)	0.099 (3)	0.000	0.015 (2)	0.000
O5	0.100 (3)	0.161 (4)	0.077 (3)	0.000	0.030 (2)	0.000
C1	0.052 (3)	0.074 (3)	0.054 (3)	0.000	0.000 (2)	0.000
C2	0.053 (3)	0.076 (3)	0.050 (2)	0.000	−0.003 (2)	0.000
C3	0.055 (3)	0.076 (3)	0.061 (3)	0.000	−0.006 (2)	0.000
C4	0.055 (3)	0.057 (3)	0.065 (3)	0.000	0.008 (2)	0.000
C5	0.074 (3)	0.069 (3)	0.047 (3)	0.000	0.004 (2)	0.000
C6	0.062 (3)	0.082 (3)	0.054 (3)	0.000	−0.007 (2)	0.000
C7	0.049 (3)	0.110 (4)	0.048 (3)	0.000	0.000 (2)	0.000
C8	0.085 (3)	0.120 (4)	0.083 (3)	0.007 (3)	−0.004 (2)	0.016 (3)
C9	0.089 (4)	0.209 (7)	0.091 (3)	0.044 (4)	−0.003 (3)	0.042 (4)
C10	0.055 (4)	0.299 (14)	0.063 (4)	0.000	−0.006 (3)	0.000

### Geometric parameters (Å, °)

**Table:**

N1—O2^i^	1.137 (6)	C3—C4	1.365 (6)
N1—O2	1.137 (6)	C3—H3	0.9300
N1—O3	1.253 (6)	C4—C5	1.373 (6)
N1—O3^i^	1.253 (6)	C5—C6	1.373 (6)
N1—C2	1.459 (6)	C5—H5	0.9300
N2—O5	1.202 (6)	C6—H6	0.9300
N2—O4	1.204 (6)	C7—C8^i^	1.358 (5)
N2—C4	1.465 (6)	C7—C8	1.358 (5)
O1—C1	1.351 (5)	C8—C9	1.375 (5)
O1—C7	1.397 (5)	C8—H8	0.9300
O2—O2^i^	0.78 (2)	C9—C10	1.360 (6)
O3—O3^i^	1.278 (15)	C9—H9	0.9300
C1—C2	1.389 (6)	C10—C9^i^	1.360 (6)
C1—C6	1.391 (6)	C10—H10	0.9300
C2—C3	1.371 (6)		
			
O2^i^—N1—O3	99.5 (6)	C3—C4—C5	122.0 (4)
O2—N1—O3	121.1 (6)	C3—C4—N2	118.3 (4)
O2^i^—N1—O3^i^	121.1 (6)	C5—C4—N2	119.6 (4)
O2—N1—O3^i^	99.5 (6)	C6—C5—C4	119.4 (4)
O2^i^—N1—C2	123.4 (4)	C6—C5—H5	120.3
O2—N1—C2	123.4 (4)	C4—C5—H5	120.3
O3—N1—C2	114.8 (4)	C5—C6—C1	120.2 (4)
O3^i^—N1—C2	114.8 (4)	C5—C6—H6	119.9
O5—N2—O4	123.1 (5)	C1—C6—H6	119.9
O5—N2—C4	118.1 (5)	C8^i^—C7—C8	123.1 (5)
O4—N2—C4	118.8 (5)	C8^i^—C7—O1	118.4 (3)
C1—O1—C7	119.5 (4)	C8—C7—O1	118.4 (3)
O1—C1—C2	117.9 (4)	C7—C8—C9	117.7 (5)
O1—C1—C6	123.7 (4)	C7—C8—H8	121.1
C2—C1—C6	118.4 (4)	C9—C8—H8	121.1
C3—C2—C1	121.6 (4)	C10—C9—C8	120.7 (6)
C3—C2—N1	117.1 (4)	C10—C9—H9	119.7
C1—C2—N1	121.3 (4)	C8—C9—H9	119.7
C4—C3—C2	118.3 (4)	C9—C10—C9^i^	120.0 (7)
C4—C3—H3	120.9	C9—C10—H10	120.0
C2—C3—H3	120.9	C9^i^—C10—H10	120.0

Symmetry codes: (i) *x*, −*y*+1/2, *z*.

### Hydrogen-bond geometry (Å, °)

**Table:**

*D*—H···*A*	*D*—H	H···*A*	*D*···*A*	*D*—H···*A*
C5—H5···O3^ii^	0.93	2.69	3.593 (8)	163
C9—H9···O2^iii^	0.93	2.50	3.274 (8)	141

Symmetry codes: (ii) *x*, *y*, *z*−1; (iii) −*x*+1/2, −*y*+1, *z*−1/2.

## References

[bb1] Gopal, R., Chandler, W. D. & Robertson, B. E. (1980). *Can. J. Chem* **58**, 658–663.

[bb2] Paul, S. & Gupta, M. (2004). *Tetrahedron Lett* **45**, 8825–8829.

[bb3] Sheldrick, G. M. (1996). *SADABS* University of Göttingen, Germany.

[bb4] Sheldrick, G. M. (2008). *Acta Cryst.* A**64**, 112–122. 10.1107/S0108767307043930 18156677

[bb5] Siemens (1996). *SMART* and *SAINT* Siemens Analytical X-ray Instruments Inc., Madison, Wisconsin, USA.

[bb6] Williamson, W. A. (1852). *J. Chem. Soc.* pp. 229–239.

